# Proteinuria is risk factor for abdominal aortic aneurysm: a nationwide population-based study

**DOI:** 10.1186/s12889-025-22989-6

**Published:** 2025-05-23

**Authors:** Kang Woong Jun, Ju-hwan Yoo, Jin-Hyung Jung, Kyung-jai Ko, Hyung-jin Cho, Mi-hyeong Kim, Kyung-do Han, Jeong-kye Hwang

**Affiliations:** 1 Division of Vascular and Transplant Surgery, Department of Surgery, Bucheon St. Mary’s Hospital, College of Medicine, The Catholic University of Korea, 327, Sosa-ro, Bucheon-si, Gyeonggi-do 14647 Republic of Korea; 2 Department of Biomedicine and Health Science, College of Medicine, The Catholic University of Korea, 222, Banpo-daero, Seocho-gu, Seoul, 06591 Republic of Korea; 3 Samsung Biomedical Research Institute, School of Medicine, Sungkyunkwan University, 2066 Seobu-ro, Jangan-gu, Suwon-si, Gyeonggi-do 16419 Republic of Korea; 4 Department of Surgery, Kangdong Sacred Heart Hospital, 150, Seongan-ro, Gangdong-gu, Seoul, 05355 Republic of Korea; 5 Division of Vascular and Transplant Surgery, Department of Surgery, Eunpyeong St. Mary’s Hospital, College of Medicine, The Catholic University of Korea, 1021, Tongil-ro, Eunpyeong-gu, Seoul, 1021, 03312 Republic of Korea; 6 Department of Statistics and Actuarial Science, Soongsil University, 369 Sangdo‑ro, Dongjak‑gu, Seoul, 06978 Republic of Korea

**Keywords:** Abdominal aortic aneurysm, Proteinuria, Chronic kidney disease, Urinalysis

## Abstract

**Background:**

Proteinuria is a well-known risk factor for cardiovascular disease. However, the impact of proteinuria on abdominal aortic aneurysm (AAA) remains unclear. In this study, we aimed to investigate the association between proteinuria and AAA. To assess the correlation between proteinuria measured by the urine dipstick test (UDT) and the risk of AAA by using nationwide population cohort data.

**Methods:**

This retrospective cohort study used data from the Korean National Health Insurance database for individuals who had health check-ups in 2009. Incident AAA was ascertained through the end of 2019. The study population was classified into the following five groups based on the UDT result: negative; trace; 1+; 2+; and ≥ 3+. The primary endpoint was newly developed AAA during the study period.

**Results:**

A total of 9,938,329 patients were enrolled. During a median follow-up of 9.3 years (interquartile range 9.1–9.6), 20,760 (0.2%) AAA events were identified, and the incidence rates of AAA were 2.21, 2.55, 4.20, 5.77 and 7.49 per 10,000 per year in the negative, trace, 1+, 2 + and 3 + ≤ proteinuria groups, respectively, compared with those without proteinuria (*P* <.001). There was a positive correlation between the degree of proteinuria and the risk of AAA, which was consistent regardless of all estimated glomerular filtration rate categories.

**Conclusion:**

Proteinuria as measured by the UDT was strongly associated with AAA and acted as an independent risk factor. These findings suggest that proteinuria may serve as a potential marker for identifying individuals at higher risk of AAA.

**Supplementary Information:**

The online version contains supplementary material available at 10.1186/s12889-025-22989-6.

## Introduction

Abdominal aortic aneurysm (AAA) is usually asymptomatic and frequently diagnosed incidentally by abdominal computed tomography when investigating unrelated abdominal symptoms or through ultrasonography screening programs for AAA. For a ruptured AAA, which is life-threatening, the mortality rates are estimated to be 80–90% for those who never reach the hospital, and the historical mortality rate of open surgical repair was approximately 50% for those who presented to a hospital [1]. Recently, with improved multiteam departments, prompt diagnoses and the development of endovascular techniques and devices, immediate survival rates are estimated to be 20–35% [2]. Nevertheless, AAA is still an issue due to its mortality and morbidity, and identifying additional risk factors for AAA may be important to reduce AAA-related mortality by surveilling high-risk patients.

General risk factors for AAA are smoking, male sex, aging, Caucasian ethnicity, hypertension and a family history of AAA [3]. These risk factors often overlap with many of the classical risk factors for atherosclerosis. Recently, chronic kidney disease (CKD) has also been recognized as a risk factor that promotes atherosclerosis as well as cardiovascular disease (CVD). Both the decline in glomerular filtration rate (GFR) and the increase in urinary protein excretion are independent risk factors for CVD.

As proteinuria is a key marker of renal damage, which is often detected earlier than a decrease in GFR, it is a predictor of cardiovascular morbidity and mortality, independent of conventional risk factors among individuals with or without hypertension or diabetes mellitus (DM) [4]. In addition, several studies have reported that proteinuria itself is a risk factor for CVD, independent of conventional risk factors, including CKD [4, 5]. Based on these results, we hypothesize that there might be an association between proteinuria, which is the key marker of CKD, and AAA, which is a subtype of CVD. Therefore, this study was conducted to identify whether the degree of proteinuria detected by the urine dipstick test (UDT) is a risk factor for AAA by using a large population-based database including nearly 10 million adults.

## Methods

The present study was a parallel study with a previous study by Jun et al. [6], “Chronic kidney disease as a risk factor for abdominal aortic aneurysm: a nationwide population-based study”. The two studies had similarities in the data source, patient group selection method, variable definitions, and statistical method.

This study was exempted by the Institutional Review Board of The Catholic University of Korea, Bucheon St. Mary’s Hospital, Seoul, Korea (HC21ZISI0121).

### Data source

The National Health Insurance Service (NHIS) is a single-payer universal coverage health insurance system for the entire Korean population managed by the Korean government. The system has 2 major health care programs: national health insurance and medical aid. Approximately 97% of the population is covered under national health insurance, and the remaining 3% is covered under medical aid. The NHIS contains all information claimed by medical service providers in Korea about diagnoses, prescriptions, and consultations [7].

### Study population and design

**Fig. 1:**
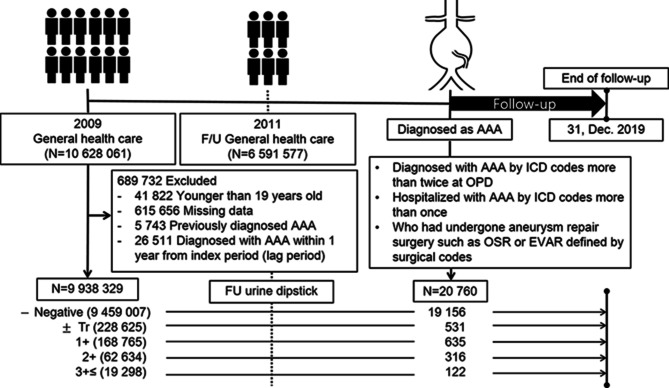
Flowchart of the study design and population. AAA, abdominal aortic aneurysm; Tr, trace; FU, follow-up; ICD, International Classification of Diseases; OSR, open surgical repair; EVAR, endovascular aneurysmal repair

We evaluated data for Koreans older than 19 years who received at least one health checkup provided by the NHIS from January 2009 to December 2009. To eliminate the possibility of including AAA patients in the baseline cohort, i) those who were diagnosed with AAA before the health care checkup (index date) were excluded. Moreover, to reduce selection bias, ii) those who were diagnosed with AAA within 1 year after the index date (lag period) were also excluded, as diseases that were thus far undiagnosed may be uncovered upon medical checkup. However, we acknowledge that due to the often asymptomatic nature of AAA, some participants might have had undiagnosed aneurysms at baseline despite these precautions. iii) Individuals with missing data for any variables (Supplementary Table 1) and those who did not receive a general health checkup in the previous 2 years were also excluded. Finally, this cohort was longitudinally followed-up from the index date until 31 December 2019 (last follow-up date) (Fig. 1).

### Definitions of abdominal aortic aneurysm and covariates

Baseline covariates and comorbidities were evaluated during the screening period and defined by using ICD-10-CM codes, as in our previous study [6, 8].

The primary end point was the diagnosis of AAA during the study period. Unlike the conceptual definition for AAA, an operational definition was applied in this study. AAA was defined as patients diagnosed with AAA codes (I71.3-I71.6, I71.8, and I71.9) more than twice at an outpatient department in the past year who had been hospitalized with one of these AAA codes more than once or who had undergone aneurysm repair surgery such as open surgical repair or endovascular aneurysmal repair (EVAR) defined by codes (O0223, O0224, O0234, or M6611, M6612).

Proteinuria was measured by using the UDT, and the degree of proteinuria was categorized as negative, trace, 1+, 2+, and ≥ 3+. While we acknowledge the limitations of a single dipstick measurement, this method has been validated in large epidemiological studies and offers high negative predictive value for ruling out significant proteinuria in population screening. Estimated glomerular filtration rate (eGFR) was estimated with the Modification of Diet in Renal Disease study equation, and CKD was defined as an eGFR of < 60 mL/min/1.73 m^2^. To analyze the hazard ratio of AAA based on the changes in proteinuria, we followed the 2011 UDT results with follow-up cohort data. Definitions of the covariates and the exact *ICD-10* codes used in this study are presented in Supplementary Table 2.

### Statistical analysis

Continuous variables are presented as the mean ± standard deviation, and categorical variables are presented as the number (percentage). Student’s *t* test was used to compare continuous variables. Categorical variables were compared with Fisher’s exact test or the χ^2^ test, as appropriate. The incidence rate of AAA according to the degree of proteinuria was calculated as the total number of AAA cases divided by the total person-years, and the result is expressed as the number per 10,000 person-years. Cumulative incidences of AAA according to the degree of proteinuria were compared using the Kaplan–Meier method and the log-rank test. Cox proportional hazards models were employed to evaluate the association between proteinuria according to the dipstick test and the risk of AAA, and the results are presented as hazard ratios (HRs) with 95% confidence intervals (CIs). A two-sided *P* <.05 was considered statistically significant. All statistical analyses were performed with SAS, version 9.2 (SAS Institute Inc. Cary, NC, USA).

## Results

### Baseline characteristics of the study population

A total of 9,938,329 individuals were enrolled in this study. The subjects were categorized into four groups according to the degree of proteinuria detected by the UDT: negative (*n* = 9,459,007; 95.2%), trace (*n* = 228,625; 2.3%), 1+ (*n* = 168,765; 1.7%), 2+ (*n* = 62,634; 0.6%), and ≥ 3+ (*n* = 19,298, 0.2%). The baseline characteristics of the study cohort by categories of dipstick proteinuria are summarized in Table 1. Overall, a higher degree of proteinuria correlated positively with age, body mass index (BMI), systolic blood pressure, diastolic blood pressure, waist circumference (WC), fasting glucose, low-density lipoprotein (LDL), total cholesterol, and triglycerides (TG) and correlated negatively with high-density lipoprotein (HDL) and eGFR (*p* <.001). In addition, a higher frequency of proteinuria was detected in individuals with hypertension, DM, hyperlipidemia, stroke, myocardial infarction (MI), ischemic heart disease (IHD), and chronic obstructive pulmonary disease (COPD). However, there was no linear trend between each group in the covariates of male sex, current smoking, heavy drinking, or regular exercise, even though it was statistically significant.

**Table 1:** Baseline characteristics of the study cohort

Characteristics	Urine protein concentration on dipstick test (Participants, No. (%))					*P*
	Negative (*N* = 9,459,007)	Trace (*N* = 228,625)	1+ (*N* = 168,765)	2+ (*N* = 62,634)	≥ 3+ (*N* = 19,298)	
**Age**, **mean(SD)**, **y**	46.9 (14.0)	48.8 (14.2)	51.1(14.5)	52.8 (14.4)	54.5 (13.9)	< 0.001
**Male**, **No. (%)**	5,168,340 (54.6)	123,934 (54.2)	90,832 (53.8)	34,827 (55.6)	10,966 (56.8)	< 0.001
**Smoking**, **No. (%)**						< 0.001
Nonsmoker	5,685,449 (60.1)	137,235 (60.0)	101,772 (60.3)	37,475 (59.8)	11,519 (59.7)	
Ex-smoker	1,312,177 (13.9)	33,141 (14.5)	25,465 (15.1)	10,022 (16.0)	3,251 (16.9)	
Current smoker	2,461,381 (26.0)	58,249 (25.5)	41,528 (24.6)	15,137 (24.2)	4,528 (23.5)	
**Regular Exercise.**^**§**^, **No.(%)**	1,710,819 (18.1)	43,028 (18.8)	31,783 (18.8)	11,879 (19.0)	3,515 (18.2)	< 0.001
**Comorbidity**, **No.(%)**						
DM	760,839 (8.0)	32,261 (14.1)	35,922 (21.3)	18,561 (29.6)	7,334 (38.0)	< 0.001
HTN	2,446,150 (25.9)	79,602 (34.8)	75,217 (44.6)	34,699 (55.4)	12,509 (64.8)	< 0.001
Dyslipidemia	1,602,175 (16.9)	49,727 (21.8)	44,895 (26.6)	20,697 (33.0)	8,061 (41.8)	< 0.001
Stroke	147,399 (1.6)	5,292 (2.3)	5,349 (3.2)	2,749 (4.4)	1,103 (5.7)	< 0.001
MI	38,169 (0.4)	1,250 (0.6)	1,264 (0.8)	612 (1.0)	288 (1.5)	< 0.001
IHD	181,381 (1.9)	6,371 (2.8)	6,438 (3.8)	3,264 (5.2)	1,339 (6.9)	< 0.001
COPD	534,047 (5.65)	14,199 (6.21)	11,142 (7.19)	4,966 (7.93)	1,743 (9.03)	< 0.001
**Anthropometric measures**, **mean (SD)**						
Body mass index^†^, kg/m^2^	23.7 (3.2)	24.0 (3.4)	24.3 (3. 6)	24.6 (3.7)	24.7 (3.8)	< 0.001
Waist Circumference^†^, cm	80.15 ± 9.04	81.06 ± 9.55	82.22 ± 9.84	83.29 ± 10.05	83.85 ± 10.07	< 0.001
Systolic blood pressure, mmHg	122.3 ± 14.9	124.1 ± 16.2	126.7 ± 17.3	129.3 ± 18.4	132.5 ± 19.6	< 0.001
Diastolic blood pressure, mmHg	76.2 ± 9.9	77.3 ± 10.8	78.5 ± 11.2	79.8 ± 11.7	81.0 ± 12.2	< 0.001
**Laboratory findings**, **mean (SD)**						
Glucose^†^, mg/dL	96.7 ± 22.8	101.8 ± 30.0	107.8 ± 38.2	114.1 ± 45.1	120.6 ± 51.5	< 0.001
Total Cholesterol^†^, mg/dL	194.8 ± 36.6	197.6 ± 38.6	199.7 ± 40.8	201.4 ± 43.3	206.7 ± 49.5	< 0.001
High density lipoprotein cholesterol^†^, mg/dL	56.2 ± 28.4	55.7 ± 27.6	55.1 ± 22.7	54.0 ± 23.7	53.7 ± 27.9	< 0.001
Low density lipoprotein cholesterol^†^, mg/dL	113.5 ± 38.6	115.3 ± 37.7	115.3 ± 40.6	115.5 ± 41.6	118.6 ± 45.6	< 0.001
Triglyceride ^‡^, mg/dL	112.0 (111.9–112.0)	114.5 (114.3-114.8)	123.1 (122.8-123.5)	133.7 (133.0-134.3)	145.3 (144.0-146.5)	< 0.001
eGlomerular filtration rate^†^, mL/min/1.73 m^2^	87.3 ± 28.1	85.3 ± 29.6	82.1 ± 30.5	77.5 ± 32.8	71.7 ± 33.7	< 0.001

^*^ Alcohol consumption was defined as mild (1–30 g/day) or heavy (over 30 g/day) based on a self-questionnaire

^†^Mean ± standard deviation. ^‡^Geometric means (95% confidence intervals). ^§^ At least 20 min or moderate-intensity exercise for at least 30 min at least once a week

DM, diabetes mellitus; HTN, hypertension; MI, myocardial infarction; IHD, ischemic heart disease; COPD, chronic obstructive pulmonary disease; BMI, body mass index; SBP, systolic blood pressure; DBP, diastolic blood pressure; HDL, high-density lipoprotein; LDL, low-density lipoprotein; TGs, triglycerides; eGFR, estimated glomerular filtration rate

### Incidence of AAA according to the urine protein concentration

**Fig. 2:**
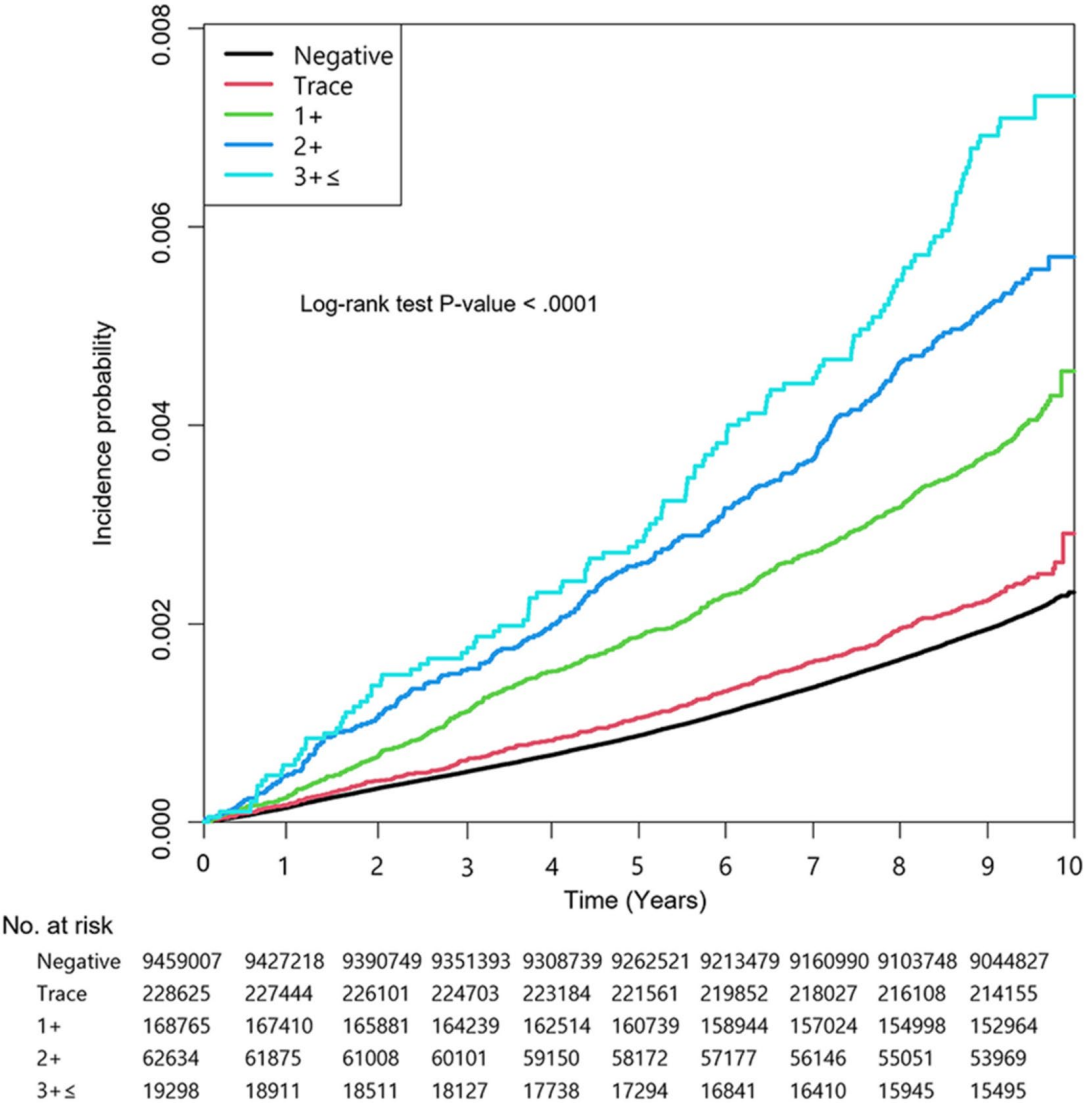
Kaplan–Meier curves for the incidence of abdominal aortic aneurysm according to the degree of dipstick proteinuria

During a median follow-up of 9.3 years (interquartile range 9.1–9.6), the incidence rates of AAA were 0.22, 0.26, 0.42, 0.57 and 0.75 per 10,000 person-years in the negative, trace, 1+, 2 + and ≥ 3 + proteinuria groups, respectively (*P* <.001). A Kaplan–Meier curve showed that the AAA incidence increased according to the degree of dipstick proteinuria (Fig. 2). A higher level of proteinuria based on the UDT was associated with an increased risk of AAA, with unadjusted HRs of 1.16 (95% CI 1.06–1.26), 1.91 (95% CI 1.77–2.07), 2.63 (95% CI 2.36–2.94) and 3.45 (95% CI 2.88–4.12) for the trace, 1+, 2 + and ≥ 3 + proteinuria groups, respectively (*P* <.001). The incidence rate of AAA in subjects with urine protein levels ≥ 3 + was nearly 1.9 times higher than that in subjects without proteinuria after adjusting for age, sex, smoking, alcohol consumption, regular exercise, DM, hypertension, hyperlipidemia, stroke, MI, and COPD. (HR 1.86, 95% CI 1.55–2.22, *P* <.001) (Table 2, Model 4). The HR was slightly attenuated after supplementing stroke, MI, COPD, BMI, eGFR, HDL, LDL, TG, and WC (HR 1.77, 95% CI 1.48–2.116, *P* <.001, Model 5) (Table 2). The results of the adjustment for all factors (Model 6) are presented in Supplementary Table 3.

**Table 2:** Incidence rates and hazard ratios of abdominal aortic aneurysm according to urine protein concentration on dipstick results adjusted for various covariates

	Urine Protein Concentration on Dipstick Test					*P*
	Negative	Trace (±)	1+	2+	≥ 3+	
**AAA events**	19,156	531	635	316	122	
**Person-years**	86,826,460.85	2,078,415.25	1,510,142.79	547,723.78	162,976.78	
**AAA incidence** ^*^ **(95% CI)**	2.21 (2.18–2.24)	2.55 (2.35–2.78)	4.20 (3.89–4.54)	5.77 (5.17–6.44)	7.49 (6.27–8.94)	< 0.001
**Model 1** ^**†**^	1	1.16 (1.06–1.26)	1.91 (1.77–2.07)	2.63 (2.36–2.94)	3.45 (2.88–4.12)	< 0.001
**Model 2** ^**†**^	1	1.00 (0.91–1.09)	1.40 (1.29–1.51)	1.73 (1.54–1.93)	2.09 (1.75–2.49)	< 0.001
**Model 3** ^**†**^	1	0.99 (0.91–1.08)	1.39 (1.28–1.50)	1.71 (1.53–1.92)	2.06 (1.72–2.46)	< 0.001
**Model 4** ^**†**^	1	0.96 (0.88–1.05)	1.32 (1.22–1.42)	1.59 (1.42–1.78)	1.86 (1.55–2.22)	< 0.001
**Model 5** ^**†**^	1	0.95 (0.87–1.04)	1.28 (1.18–1.39)	1.53 (1.37–1.71)	1.77 (1.48–2.12)	< 0.001

^*^ Per 10 000 person-years

^†^Data are presented with 95% confidence intervals

Model 1: Not adjusted for any covariates. Model 2: Adjusted for age and sex. Model 3: Model 2 with additional adjustment for smoking (nonsmoker, ex-smoker, current), alcohol consumption (none, mild, heavy), and regular exercise (none, moderate). Model 4: Model 3 with additional adjustment for diabetes mellitus, hypertension, hyperlipidemia, stroke, myocardial infarction, and chronic obstructive pulmonary disease. Model 5: Model 4 with additional adjustment for body mass index, estimated glomerular filtration rate, high-density lipoprotein, low-density lipoprotein, triglycerides, and waist circumference

Covariates were entered in the model by continuous variables for age, glomerular filtration rate, body mass index high-density lipoprotein, low-density lipoprotein, triglycerides, and waist circumference and by categorical variables for sex, smoking (nonsmoker, ex-smoker, current smoker), alcohol consumption (non, mild, heavy), regular exercise (none, regular-intensity, high-intensity), diabetes mellitus, hypertension, hyperlipidemia, stroke, myocardial infarction, and chronic obstructive pulmonary disease

AAA, abdominal aortic aneurysm; CI, confidence interval; HR, hazard ratio

### Subgroup analysis

**Fig. 3:**
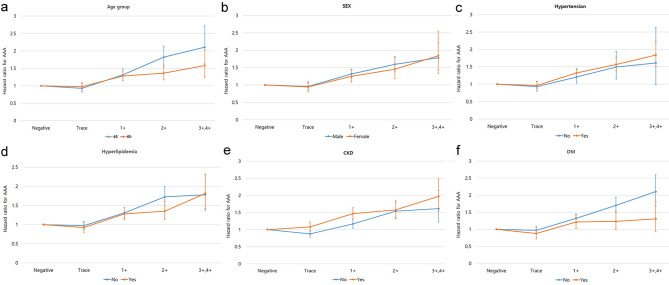
Subgroup analysis. Upward trend of the hazard ratio of abdominal aortic aneurysm according to the severity of dipstick proteinuria, regardless of (**a**) age, (**b**) sex, (**c**) hypertension, (**d**) hyperlipidemia, (**e**) chronic kidney disease, and (**f**) diabetes mellitus. Hazard ratios were calculated using a Cox proportional model adjusted for age and sex, smoking, alcohol consumption, regular exercise, diabetes mellitus, hypertension, hyperlipidemia, stroke, myocardial infarction, chronic obstructive pulmonary disease body mass index, and glomerular filtration rate. AAA, abdominal aortic aneurysm; CKD, chronic kidney disease; DM, diabetes mellitus

Univariate subgroup analysis was performed to identify the effects of proteinuria on the incident AAA according to several factors, such as age group, sex, hypertension, hyperlipidemia, DM and CKD. A proportional increase in the risk of AAA with the level of proteinuria was consistently observed, regardless of age group, sex, hypertension, hyperlipidemia, DM and CKD (Fig. 3). Furthermore, the risk of AAA was more prominent in subjects with CKD than in those without CKD in each of the positive proteinuria groups (HR 1.47, 95% CI 1.31–1.64; HR 1.58, 95% CI 1.35–1.84; and HR 1.97, 95% CI 1.56–2.48 vs. HR 1.17, 95% CI 1.04–1.3; HR 1.54, 95% CI 1.31–1.80; and HR 1.61, 95% CI 1.22–2.14, in 1+; 2+; and ≥ 3+, respectively) (Fig. 3e). On the other hand, the risk of AAA was significantly lower in individuals with DM in all dipstick-positive proteinuria groups than in those without DM (HR 1.21, 95% CI 1.03–1.427; HR 1.23, 95% CI 0.99–1.53; and HR 1.31, 95% CI 0.94–1.82 vs. HR 1.32, 95% CI 1.21–1.45; HR 1.70, 95% CI 1.49–1.93; and HR 2.10, 95% CI 1.70–2.60, in 1+; 2+; and ≥ 3+, respectively) (Fig. 3f).

**Fig. 4:**
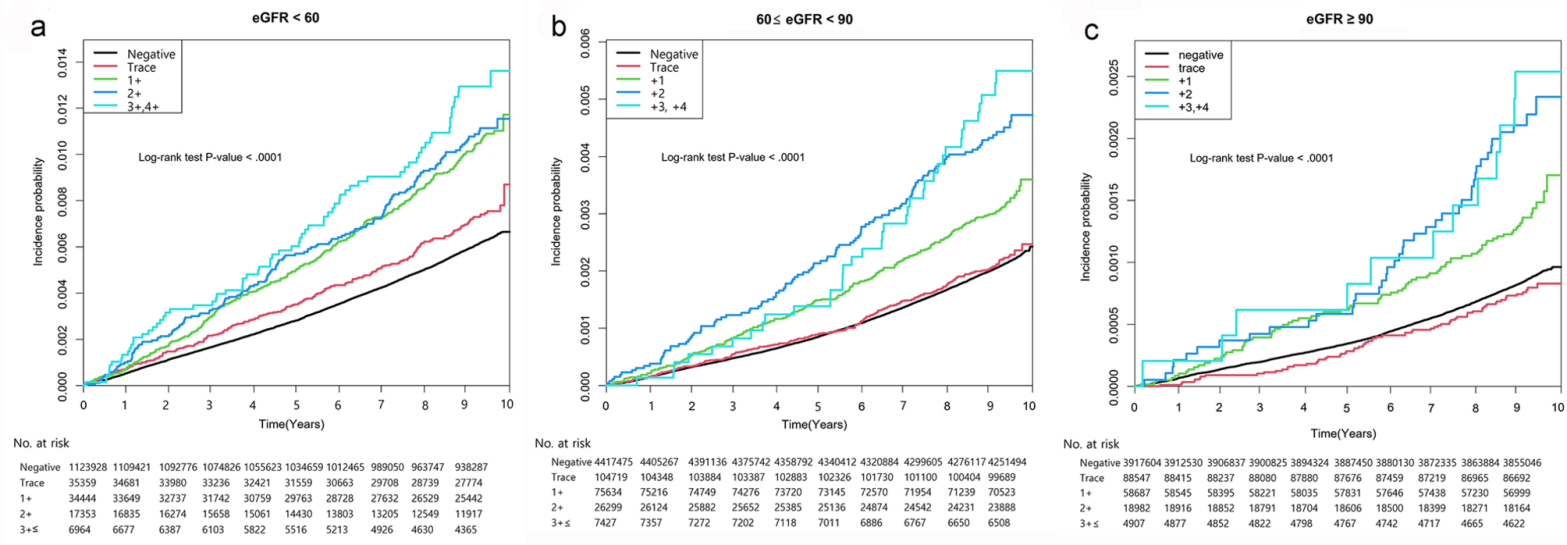
Kaplan–Meier curves for the incidence of abdominal aortic aneurysm according to the degree of dipstick proteinuria with a subgroup analysis according to the estimated glomerular filtration rate: (**a**) eGFR < 60 mL/min/1.73 m^2^, (**b**) 60 ≤ eGFR < 90 mL/min/1.73 m^2^, and (**c**) eGFR ≥ 90 mL/min/1.73 m^2^. eGFR, estimated glomerular filtration rate

To adjust for the impact of kidney function on the risk of AAA, a sensitivity test was undertaken using eGFR, which was classified as eGFR < 60 mL/min/1.73 m^2^, 60 ≤ eGFR < 90 mL/min/1.73 m^2^, and eGFR ≥ 90 mL/min/1.73 m^2^, and added to the multivariate analysis. In the subgroup analysis, there was a positive correlation between the degree of dipstick proteinuria and the risk of AAA, which was consistent regardless of all eGFR categories. However, the association between the severity of proteinuria and incident AAA was more prominent in the eGFR < 60 mL/min/1.73 m^2^ subgroup than in the 60 ≤ eGFR < 90 mL/min/1.73 m^2^ and eGFR ≥ 90 mL/min/1.73 m^2^ subgroups (*P* =.035) (Fig. 4). There were no multiple collinearities between eGFR and urine protein for which the variance inflation factor (VIF) was observed to be below 10 (VIF = 1.00097).

### Changes in urine protein based on dipstick results and the risk of AAA

To identify whether there is a difference in the risk of AAA according to alterations in the dipstick result over time, we performed subgroup analysis using two-year follow-up cohort data. We defined “(-)” as negative or trace and “(+)” as overt proteinuria (proteinuria ≥ 1+) based on the UDT and classified the subjects into one of the “non-proteinuria” ((-)→(-)), “persistent proteinuria” ((+)→(+)), “improved proteinuria” ((+)→(-)), and “new proteinuria” ((-)→(+)) groups in 2009 and 2011, respectively. Of a total of 9,938,329 individuals, 6,591,577 (66.3%) had undergone a second health care checkup within 2 years after the baseline checkup. Of these, most of the subjects (*n* = 6,337,769 (96.1%)) who initially had a urine dipstick negative or trace result also had the same result at the 2-year follow-up (non-proteinuria group). For individuals who showed positive dipstick results during follow-up (new proteinuria group, *n* = 101,591 (1.5%)), the risk of AAA increased significantly after adjustment for covariates (HR 1.25, 95% CI (1.11–1.39), Model 5, *P* <.001) (Table 3). In addition, the risk of developing AAA was highest in the persistent proteinuria group (*n* = 31,839 (0.6%)), and it increased nearly 50% (HR 1.51, 95% CI (1.29–1.77), Model 5, *P* <.001) compared with the non-proteinuria group. Nevertheless, in subjects whose dipstick proteinuria result became negative (improved proteinuria group, *n* = 120,378 (1.8%)), the risk of AAA decreased significantly compared to that in the persistent proteinuria group (from HR 1.51, 95% CI (1.29–1.77) to HR 1.28, 95% CI (1.16–1.43), Model 5, *P* <.001). The results of the adjustment for all factors (Model 6) are presented in Supplementary Table 4.

**Table 3:** Incidence rates and hazard ratios of abdominal aortic aneurysm according to changes in urine protein dipstick results over 2 years

	Changes in urine protein dipstick results (2009 -> 2011)				*P*
	Non-proteinuria ((-)-> (-))	New proteinuria ((-)-> (+))	Improved proteinuria ((+)-> (-))	Persistent proteinuria ((+)-> (+))	
**AAA events**	10,515	317	362	156	
**Person-years**	46,283,237.38	724,831.77	866,571.84	220,819.7	
**AAA incidence** ^*^ **(95% CI)**	2.27 (2,23-2.32)	4.37 (3.92–4.88)	4.20 (3.77–4.63)	7.06 (6.04–8.27)	< 0.001
**Model 1** ^**†**^	1	1.93 (1.73–2.16)	1.84 (1.66–2.05)	3.13 (2.68–3.67)	< 0.001
**Model 2** ^**†**^	1	1.36 (1.21–1.52)	1.38 (1.25–1.54)	1.79 (1.53–2.10)	< 0.001
**Model 3** ^**†**^	1	1.34 (1.20–1.49)	1.37 (1.24–1.53)	1.76 (1.50–2.06)	< 0.001
**Model 4** ^**†**^	1	1.28 (1.14–1.43)	1.31 (1.18–1.45)	1.60 (1.37–1.88)	< 0.001
**Model 5** ^**†**^	1	1.25 (1.11–1.39)	1.28 (1.16–1.43)	1.51 (1.29–1.77)	< 0.001

^*^ Per 10 000 person-years

^†^Data are presented with 95% confidence intervals

Model 1: Not adjusted for any covariates. Model 2: Adjusted for age and sex. Model 3: Model 2 with additional adjustment for smoking (nonsmoker, ex-smoker, current), alcohol consumption (none, mild, heavy), and regular exercise (none, moderate). Model 4: Model 3 with additional adjustment for diabetes mellitus, hypertension, hyperlipidemia, stroke, myocardial infarction, and chronic obstructive pulmonary disease. Model 5: Model 4 with additional adjustment for body mass index, estimated glomerular filtration rate, high-density lipoprotein, low-density lipoprotein, triglycerides, and waist circumference

Covariates were entered in the model by continuous variables for age, glomerular filtration rate, body mass index high-density lipoprotein, low-density lipoprotein, triglycerides, and waist circumference and by categorical variables for sex, smoking (nonsmoker, ex-smoker, current smoker), alcohol consumption (non, mild, heavy), regular exercise (none, regular-intensity, high-intensity), diabetes mellitus, hypertension, hyperlipidemia, stroke, myocardial infarction, and chronic obstructive pulmonary disease

AAA, abdominal aortic aneurysm; CI, confidence interval; HR, hazard ratio

## Discussion

We performed a large-scale nationwide study to identify the impact of dipstick proteinuria on the incident AAA using national health screening data for nearly 10 million people in the general population. The following results were obtained: (1) proteinuria detected by the dipstick test was significantly associated with incident AAA; (2) our analysis suggests an association between increasing severity of proteinuria and higher risk of AAA; (3) this association persisted after adjustment for several covariates, including kidney function based on eGFR; (4) the risk of AAA was decreased in DM compared with non-DM; and (5) our findings indicate that persistent proteinuria was associated with the highest risk of AAA, with risk levels varying according to changes in proteinuria status in follow-up UDT results.

The UDT is a semiquantitative assay, and it is widely used in large-scale epidemiologic studies and in screening the non-risk population in whom screening for proteinuria is not recommended by current guidelines. However, the evidence for the accuracy of dipstick urinalysis to quantify proteinuria is still conflicting, and several studies have reported that dipstick proteinuria has a low sensitivity and predictive value [9, 10]. In contrast, several studies have reported that to detect proteinuria in the general population, a negative dipstick urinalysis demonstrates a high negative predictive value (NPV), which is nearly over 95% in both albumin-to-creatinine ratios ≥ 30 mg/g and ≥ 300 mg/g [9, 11]. As the high NPV of a UDT is applied to the general population, the probability of a false negative is low/minimal, and it is inferred that the HRs presented in this study could be interpreted as an estimation of the minimal incidence risk. Furthermore, due to the high false-positive rate of dipstick proteinuria in the general population [9, 11], several previous studies have defined persistent proteinuria as more than 2 times the dipstick positive value. However, this method has been shown to lead to a decreased/lower NPV [12], and most of the general population studies utilize a single measurement to reduce the incidence of false-negative cases [13]. Thus, in the present study, we also used the single measurement results to minimize the false-negative rate.

There are several studies on the association between CKD and the incidence of AAA, but the reported results are conflicting [14–16]. Recently, a study that used data from the Atherosclerosis Risk in Communities (ARIC) study, which enrolled approximately fifteen thousand patients, demonstrated that CKD itself and its measurements, such as eGFR and microalbuminuria, are associated with the risk of AAA and greater abdominal aortic diameter [17]. On the other hand, our study enrolled nearly 10 million people. Moreover, to assess the value of dipstick urine proteinuria for incident AAA, we performed this study (1) by adjusting for renal function by including eGFR as a covariate or (2) by analyzing subgroups classified by several factors, including eGFR. We found that proteinuria is an independent risk factor for AAA regardless of CKD, and these predictive effects of proteinuria were more prominent in the low eGFR group (< 60 mL/min/1.73 m^2^) than in the 60 ≤ eGFR < 90 and eGFR ≥ 90 mL/min/1.73 m^2^ subgroups.

In the current investigation, individuals with DM exhibited a significantly reduced risk of AAA compared to those without DM in all cases of dipstick-positive proteinuria (≥ 1+). The potential protective effect against AAA has been consistently demonstrated in numerous clinical studies [18–21]. The underlying pathophysiology for protective effect of DM on AAA can be attributed to two main mechanisms: i) the hyperglycemic effect DM itself and/or ii) the direct/indirect effect of antidiabetic drugs. Recent studies lean toward the latter mechanism, considering that features associated with DM, such as increased arterial stiffness, endothelial dysfunction and arterial calcifications, are recognized risk factors of CVD and likely contribute to some extent to the development of AAA [22]. Conversely, various oral antidiabetic drugs, such as biguanides [23], thiazolidinediones [24, 25], α-glucosidase inhibitors [26], dipeptidyl peptidase-4 (DPP-4) inhibitor [27], Type-2 sodium-glucose co-transporter (SLGT) inhibitors [28] etc., have demonstrated effect against inflammatory, oxidative stress, elastin degradation, proteolysis, and arterial calcification [22]. These mechanisms probably contribute to protective effect against AAA development and progression. While this study did not reveal a definite protective effect of DM on AAA, the risk was prominently lower in individual with DM compared to those without, further clinical studies are warranted to identify the precise underlying mechanism.

Interestingly, we found that the risk of AAA increased or decreased according to the changes in follow-up UDT results. The Reduction in Endpoints in Non-insulin-dependent DM with the Angiotensin II Antagonist Losartan (RENAAL) study demonstrated the renal-protective effect of losartan in patients with diabetic nephropathy, which resulted in the reduction of proteinuria and an improvement in cardiovascular outcomes [29]. In our study, the risk of AAA increased nearly 25% in individuals with new-onset proteinuria after two years of follow-up compared to the non-proteinuria group; in contrast, conversion to a negative result (improved proteinuria) between serial dipstick tests was associated with a nearly 23% reduction in AAA risk compared to the persistent proteinuria group, even after adjusting for covariates (Model 5).

There is no clear explanation for how proteinuria impacts the incidence of AAA. However, several plausible hypothetical mechanisms exist, with one aspect being that proteinuria serves as a key marker of CKD. First, both CKD and AAA share well-established risk factors and exhibit an increased risk of CVD, indicating that the risk factors associated with CKD could influence the development of AAA. Second, the arteries of CKD patients exhibit atherosclerosis, calcifications, and increased stiffness compared to healthy populations. The atherosclerotic plaque in the artery of CKD patients has been shown to be deficient in collagen fibers and increased in calcium deposition, potentially creating conditions conducive to aneurysmal degeneration [10]. Third, various matrix metalloproteinases (MMPs), including MMPs 2, 8, and 9, the A Disintegrin and Metalloproteinase (ADAM) family, and the tissue inhibitors of metalloproteinases (TIMPs), are concurrently implicated in the development of both aortic aneurysms and CKD [30]. If MMP- 2, 8, 9, and TIMP are overexpressed and hyperactivated in the aneurysm simultaneously, these MMPs may damage tubular cells and activate a cascade of proinflammatory and profibrotic signals in the kidney, resulting in accelerated CKD progression [30, 32]. Fourth, from a biomechanical perspective, the AAA wall in CKD patients exhibits a reduced “failure tension” (defined as the resistance of a wall portion against rupture, independent of the actual wall thickness) [32]. This suggests that the diminished content of collagenous fibers in CKD patients may contribute to the reduction in the failure tension of the AAA wall, which might increase the risk of AAA and its rupture rate [32].

### Limitations

This study has several limitations. First, this study relies on ICD codes for AAA identification, which may underestimate the prevalence of asymptomatic AAA at baseline. We applied a one-year lag period after the index date to minimize this potential misclassification, but we acknowledge that some participants might have had undiagnosed AAA at baseline. Second, the retrospective observational design of this study restricts the interpretation of a causal relationship. Third due to the utilization of claims data, disease codes might not accurately represent the participants’ specific disease factors, such as aneurysmal size, symptoms (e.g., asymptomatic, pain or ruptures), family history of aortic arch, and genetic syndrome, for which data were unavailable. And the NHIS database structure presents technical constraints for integrating individual-level clinical data with diagnostic codes, potentially affecting the precision of disease definitions. This is particularly relevant when defining chronic conditions such as hypertension, diabetes, and CKD, which may influence the interpretation of our findings regarding the association between proteinuria and AAA. Fourth, a baseline UDT result was obtained by a single measurement, which may lead to random measurement errors and regression dilution bias [33]. Due to these biases, the actual association between proteinuria and incident AAA may be underestimated. Fifth, as proteinuria is detected by dipstick urinalysis, which is a semi-quantitative assay and relatively insensitive, it is not registered as positive until total protein excretion is more than approximately 300 mg per day. As a result, the interpretation of the results obtained from these tests might be controversial. Sixth, prescription drug use (angiotensin-converting enzyme inhibitors (ACEis), angiotensin receptor blockers (ARBs), and statins) might affect proteinuria and renal function. Because many patients with proteinuria and hypertension are often treated with ACEIs and/or ARBs, there might be confounders. Seventh, our study population was limited to health checkup participants, potentially excluding those with limited healthcare access or severe disease who avoid checkups. Despite the Korean screening program’s broad coverage, participation varies and individuals with poorer health might be underrepresented. Additionally, surveillance bias exists as proteinuria patients likely receive more medical attention and imaging studies, potentially increasing AAA detection compared to those without proteinuria, which could have influenced our findings. Finally, this study cohort was entirely composed of a Korean population, and the results may not be generalizable to all ethnic groups.

### Strengths

Nonetheless, this study result demonstrates the presence of a dose‒response relationship between the degree of proteinuria and AAA risk, coupled with the follow-up of proteinuria in the absence of AAA at baseline with a lag period of 1 year by eliminating selection bias. Since the national health insurance corporation is a single insurer that manages the NHIS in Korea, virtually all Koreans (approximately 50 million) are enrolled in this program; therefore, the NHIS data could represent the entire Korean population. On this basis, this is the largest population study that has demonstrated the association between the degree of proteinuria and AAA, and by adjusting for the well-known risk factors for AAA, we increased the robustness of the study.

## Conclusions

Proteinuria as measured by the UDT was strongly associated with AAA and acted as an independent risk factor. Even trace proteinuria was associated with elevated AAA risk, and this risk showed an upward trend according to the severity of dipstick proteinuria. The risk of AAA increased as the result of dipstick proteinuria increased from negative to positive, and the risk decreased as proteinuria improved at the 2-year follow-up. While these findings suggest an association between proteinuria and AAA risk, the clinical utility of using proteinuria measurements for AAA risk stratification requires validation in prospective studies with more precise proteinuria measurements and comprehensive imaging assessment of AAA. Future research should specifically address whether different thresholds of proteinuria warrant consideration in AAA screening algorithms, and whether the addition of proteinuria to existing risk models improves their predictive value. Further prospective investigations using a 24-hour urine protein test or an albumin-to-creatinine ratio (ACR)/ protein-to-creatinine ratio (PCR) test to identify the pathophysiology between proteinuria and AAA development.

## Electronic supplementary material

Below is the link to the electronic supplementary material.

Supplementary Material 1: Additional file: Supplementary Table 1: Missing data categorized by variables. Supplementary Table 2: International Classification of disease (ICD) codes 10 used in the study for identifying patients’ baseline comorbidities. Supplementary Table 3: Incidence rates and hazard ratios of abdominal aortic aneurysm according to urine protein concentration on dipstick results adjusted for all covariates.Supplementary Table 4: Incidence rates and hazard ratios of abdominal aortic aneurysm according to changes in urine protein dipstick results over 2 years.

## Data Availability

No datasets were generated or analysed during the current study.

## Abbreviations

AAA: Abdominal aortic aneurysms
ACEis: Angiotensin-converting enzyme inhibitors
ACR: Albumin-to-creatinine ratio
ADAM: A disintegrin and metalloproteinase
ARBs: Angiotensin receptor blockers
ARIC: Atherosclerosis Risk in Communities
BMI: Body mass index
CI: Confidence interval
CKD: Chronic kidney disease
CVD: Cardiovascular disease
DM: Diabetes mellitus
eGFR: Estimated glomerular filtration rate
HR: Hazard ratio
MMPs: Matrix metalloproteinases
NHIS: National Health Insurance Service
NPV: Negative predictive value
PCR: Protein-to-creatinine ratio
TIMPs: Tissue inhibitors of metalloproteinases
UDT: Urine dipstick test
VIF: Variance inflation factor
WC: Waist circumference
