# Supplementary material for: Proteinuria is risk factor for abdominal aortic aneurysm: a nationwide population-based study
Source: BMC Public Health. 2025 May 23;25:1897. doi: 10.1186/s12889-025-22989-6 (PMC12100979; doi:10.1186/s12889-025-22989-6)
Supplement: Supplementary file 1 — Supplementary Material 1: Additional file: Supplementary Table 1: Missing data categorized by variables. Supplementary Table 2: International Classification of disease (ICD) codes 10 used in the study for identifying patients’ baseline comorbidities. Supplementary Table 3: Incidence rates and hazard ratios of abdominal aortic aneurysm according to urine protein concentration on dipstick results adjusted for all covariates.Supplementary Table 4: Incidence rates and hazard ratios of abdominal aortic aneurysm according to changes in urine protein dipstick results over 2 years. [file 12889_2025_22989_MOESM1_ESM.docx]

**Supplementary Table 1.** Missing data categorized by variables

| **Variables** | **Missing N** |
| --- | --- |
| Smoking | 189,197 |
| Alcohol consumption | 164,940 |
| Physical activity(exercise) | 184,183 |
| eGFR | 20,948 |
| BMI | 6,513 |
| Waist Circumference | 6,149 |
| Glucose | 3,391 |
| SBP | 2,836 |
| DBP | 2,852 |
| TG | 9,757 |
| HDL | 4,707 |
| LDL | 39,191 |
| Urine protein | 39,530 |
| **Total number** | **615,656** |

eGFR, estimated glomerular filtration rate.; BMI, body mass index; SBP, systolic blood pressure; DBP, diastolic blood pressure; TG, triglycerides; HDL, high-density lipoprotein; LDL, low-density lipoprotein

**Supplementary Table 2.** International Classification of disease (ICD) codes 10 used in the study for identifying patients’ baseline comorbidities

| **Variable** | **ICD-10 code** | **Definition** |
| --- | --- | --- |
| AAA | I71, I713-716, I718, or I719  Surgery code  O0223, O0224, O0234, or M6611, M6612 | At least 2 claims per year under ICD-10 codes, or at least one claim for hospitalization under the same ICD-10 codes, or at least one claim for surgery under the same ICD-10 codes |
| Hypertension | I10-15 | One diagnosis during hospitalization or more than twice at outpatient clinics for the past year |
| Diabetes Mellitus | E11-14 | At least 1 claim for the prescription of antidiabetic medication or insulin under ICD-10 codes |
| Dyslipidemia | E78 | At least 1 claim for the prescription of hyperlipidemia medication under ICD-10 code |
| Ischemic heart disease | I20-25 | At least 1 claim during hospitalization or more than twice at outpatient clinics over the past year under *ICD-10* code |
| Myocardial infarction | I21; I22 | A stated history of MI or one diagnosis during hospitalization or more than twice at outpatient clinics over the past three years |
| Stroke | I63; I64 | At least 1 claim under *ICD-10* codes or stated history of stroke by the patient |
| COPD | J43; J44 | At least one diagnosis over the past three years under *ICD-10* codes |

Abbreviations: AAA, abdominal aortic aneurysm; COPD, chronic obstructive pulmonary disease; ICD-10, International Classification of Diseases-Tenth Revision

<Definition of covariates>

Current smokers were defined as those who smoked 100 or more cigarettes in their life and continued smoking within 1 month before the 2009 nationwide health checkup, and ex-smokers were defined as those who stopped smoking for more than 1 month before the checkup.

Alcohol consumption was classified into 3 grades according to the amount of daily use, namely, none, mild to moderate (1–30 g/day), and heavy (over 30 g/day) based on a self-questionnaire.

Regular exercise was defined as high-intensity exercise for at least 20 min or moderate-intensity exercise for at least 30 min at least once a week based on a self-report questionnaire

**Supplementary Table 3.** Incidence rates and hazard ratios of abdominal aortic aneurysm according to urine protein concentration on dipstick results adjusted for all covariates

|  | **Urine Protein Concentration on Dipstick Test** | | | | | **p** |
| --- | --- | --- | --- | --- | --- | --- |
|  | **Negative** | **Trace (±)** | **1+** | **2+** | **3+≤** |  |
| AAA events | 19,156 | 531 | 635 | 316 | 122 |  |
| Person-years | 86,826,460.85 | 2 ,078,415.25 | 1,510,142.79 | 547,723.78 | 162,976.78 |  |
| AAA incidence^a^ (95% CI) | 2.21  (2.18-2.24) | 2.55  (2.35-2.78) | 4.20  (3.89-4.54) | 5.77  (5.17-6.44) | 7.49  (6.27-8.94) | <.001 |
| Model 6^b^ | 1 | 0.96  (0.87-1.04) | 1.30  (1.20-1.41) | 1.56  (1.37-1.71) | 1.82  (1.52-2.18) | <.001 |

^a^Per 10,000 person-years.

^b^Data are presented with (95% confidence intervals).

Model 6: Model 6 with glucose, systolic blood pressure, and diastolic blood pressure

Covariates were entered in the model by continuous variables for age, glomerular filtration rate, body mass index high-density lipoprotein, low-density lipoprotein, triglycerides, and waist circumference and by categorical variables for sex, smoking (nonsmoker, ex-smoker, current smoker), alcohol consumption (non, mild, heavy), regular exercise (none, regular-intensity, high-intensity), diabetes mellitus, hypertension, hyperlipidemia, stroke, myocardial infarction, and chronic obstructive pulmonary disease.

AAA, abdominal aortic aneurysm; CI, confidence interval; HR, hazard ratio.

**Supplementary Table 4.** Incidence rates and hazard ratios of abdominal aortic aneurysm according to changes in urine protein dipstick results over 2 years

|  | **Changes in urine protein dipstick results (2009 -> 2011)** | | | | **p** |
| --- | --- | --- | --- | --- | --- |
|  | **Non-proteinuria ((-)-> (-))** | **New proteinuria ((-)-> (+))** | **Improved proteinuria**  **((+)-> (-))** | **Persistent proteinuria**  **((+)-> (+))** |  |
| AAA events | 10,515 | 317 | 362 | 156 |  |
| Person-years | 46,283,237.38 | 724,831.77 | 866,571.84 | 220,819.7 |  |
| AAA incidence^a^  (95% CI) | 2.27 (2,23-2.32) | 4.37 (3.92-4.88) | 4.20 (3.77-4.63) | 7.06 (6.04-8.27) | <.001 |
| Model 6 ^b^ | 1 | 1.27 (1.13-1.42) | 1.29 (1.16-1.44) | 1.55 (1.32-1.81) | <.001 |

^a^Per 10,000 person-years.

^b^Data are presented with 95% confidence intervals).

Model 6: Model 5 with glucose, systolic blood pressure, and diastolic blood pressure

Covariates were entered in the model by continuous variables for age, glomerular filtration rate, body mass index high-density lipoprotein, low-density lipoprotein, triglycerides, and waist circumference and by categorical variables for sex, smoking (nonsmoker, ex-smoker, current smoker), alcohol consumption (non, mild, heavy), regular exercise (none, regular-intensity, high-intensity), diabetes mellitus, hypertension, hyperlipidemia, stroke, myocardial infarction, and chronic obstructive pulmonary disease.

AAA, abdominal aortic aneurysm; CI, confidence interval; HR, hazard ratio.
